# Efficacy and Budget Impact of a Tailored Psychological Intervention Program Targeting Cancer Patients With Adjustment Disorder: A Randomised Controlled Trial

**DOI:** 10.1002/pon.70123

**Published:** 2025-03-15

**Authors:** K. Holtmaat, F. E. van Beek, L. M. A. Wijnhoven, J. A. E. Custers, E. J. Aukema, S. E. J. Eerenstein, I. M. van Oort, J. E. M. Werner, J. A. Wegdam, I. L. E. Jansen‐Engelen, I. H. J. T. de Hingh, S. Verheul, D. T. van der Beek, G. Wekking, I. Steggerda, V. M. H. Coupé, N. Horevoorts, A. M. de Korte, C. Lammens, B. I. Lissenberg‐Witte, B. H. de Rooij, J. B. Prins, I. M. Verdonck‐de Leeuw, F. Jansen

**Affiliations:** ^1^ Department of Clinical, Neuro and Developmental Psychology Vrije Universiteit Amsterdam Amsterdam the Netherlands; ^2^ Treatment and Quality of Life Cancer Center Amsterdam Amsterdam the Netherlands; ^3^ Amsterdam Public Health, Mental Health Amsterdam the Netherlands; ^4^ Department of Medical Psychology Research Institute for Medical Innovation Radboudumc Nijmegen Nijmegen the Netherlands; ^5^ Centre for Psycho‐Oncology Ingeborg Douwes Centrum Amsterdam the Netherlands; ^6^ Department of Otolaryngology‐Head and Neck Surgery Amsterdam UMC Vrije Universiteit Amsterdam Amsterdam the Netherlands; ^7^ Department of Urology Radboudumc Nijmegen Nijmegen the Netherlands; ^8^ Department of Surgery Oncology Radboudumc Nijmegen Nijmegen the Netherlands; ^9^ Department of Surgery Elkerliek Hospital Helmond the Netherlands; ^10^ Department of Surgery St. Jans Gasthuis Hospital Weert the Netherlands; ^11^ Department of Surgery Catharina Hospital Eindhoven the Netherlands; ^12^ Department of Medical Psychology CWZ Nijmegen Nijmegen the Netherlands; ^13^ Department of Psychology and Psychiatry Haga Hospital The Hague the Netherlands; ^14^ Psychologists Practice Wekking Harderwijk the Netherlands; ^15^ Bloem Psychology Voorburg the Netherlands; ^16^ Department of Epidemiology and Data Science Amsterdam UMC Vrije Universiteit Amsterdam Amsterdam the Netherlands; ^17^ Department of Medical and Clinical Psychology Tilburg University Tilburg the Netherlands; ^18^ The Netherlands Comprehensive Cancer Organization Utrecht the Netherlands

**Keywords:** adjustment disorder, anxiety, cancer, cost‐benefit analysis, depression, health‐related quality of life, oncology, psychological distress, psychological intervention, randomised controlled trial

## Abstract

**Background:**

Evidence on the efficacy of psychological interventions targeting cancer patients diagnosed with an adjustment disorder is scarce.

**Aims:**

This study aimed to investigate the efficacy and budget impact of a tailored psychological intervention program (AD‐program) targeting cancer patients with adjustment disorder (AD).

**Methods:**

Patients (*n* = 59) were randomised to the intervention or control group. The AD‐program consisted of three modules: psychoeducation (1–4 sessions) and two additional modules (maximum of 6 sessions per module) provided when needed. The primary outcome was psychological distress (HADS). Secondary outcomes were mental adjustment to cancer (MAC) and health‐related quality of life (EORTC QLQ‐C30). Measures were completed at baseline and 3 and 6 months after randomisation. The budget impact analyses were based on the population size, the costs of the AD‐program, and other costs potentially affected by the AD‐program.

**Results:**

The mean psychological distress score in the intervention group (*n* = 33) decreased over time (*M* = 19.2 at T0, *M* = 15.6 at T6). This decrease was not significantly different from decrease in the control condition (*n* = 26, *M* = 17.5 at T0, *M* = 15.9 at T6, *p* > 0.05). Also, there were no significant differences between the two conditions on the secondary outcomes. The budget impact of the AD‐program was estimated at 7–28 million euros per year (to treat 14,430 patients).

**Conclusions:**

The effect of the AD‐program was not statistically significant in this RCT. Limitations include that this study was underpowered due to recruitment difficulties during the COVID‐19 pandemic. More research on the efficacy and implementation of the AD‐program is warranted.

**Trial Registration:**

Netherlands Trial Register identifier: NL7763. Registered on 3 June 2019

## 1 Background

Adjustment disorder (AD) is defined by clinically significant emotional or behavioural symptoms in response to an identifiable stressor [1, 2]. These symptoms are associated with significant impairment in important areas of functioning and they do not meet the criteria for another mental disorder [1]. Cancer typically involves a series of distressing experiences over an extended period of time [2]. In cancer patients, the prevalence of AD is estimated to be 3%–19% [3, 4, 5, 6]. The majority (65%) of cancer patients with AD accept referral to a psychological intervention, when such intervention is offered [4, 7]. Several recent systematic reviews and meta‐analyses provided evidence for the effectiveness of psychological interventions in cancer patients in reducing symptoms of anxiety, depression, distress, and fatigue, or improving health‐related quality of life [8, 9, 10, 11, 12, 13, 14, 15, 16, 17]. Systematic reviews also showed that psychological interventions are likely to be cost‐effective [18, 19].

In 2016, a national guideline was developed in the Netherlands on the diagnosis and treatment targeting AD in cancer patients [2]. This guideline recommended to screen for need for psychological care throughout the cancer trajectory and to offer a diagnostic interview in case of indications of AD. This interview should focus on interactions between stressors, resilience and symptoms [20]. The guideline recommended to start with psychoeducation. If needed, this could be followed by a variety of psychological interventions specifically developed for cancer patients or from regular mental health care, tailored to the patient's needs, functional limitations, illness phase and prognosis. Based on this guideline, the AD‐program was designed, consisting of three modules provided by a healthcare psychologist: first a psychoeducation module of 1–4 sessions, followed, if needed, by two additional modules (each 1–6 sessions) of tailored psychological interventions [21].

Interventions offered in the AD‐program are evidence‐based, but evidence specifically targeting cancer patients diagnosed with AD is scarce. Furthermore, the AD‐program encompasses a personalised and integrated approach to AD in cancer patients, from psychoeducation to a variety of tailored interventions. Evidence for such a program, which reflects the way psycho‐oncological care is typically offered in the real world, is limited. Evidence on the cost‐effectiveness is even more scarce. An observational real world pre‐post study among 563 cancer patients showed a beneficial effect of the AD‐program on distress, fatigue, insomnia and health‐related quality of life. The participants attended, on average, 10 sessions. The average cost per treatment was estimated at €1.141 (based on prices of 2018–2021) [22]. A randomised controlled trial (RCT) is needed to confirm these encouraging findings and to obtain insight into the budget impact, that is, the financial consequences from a healthcare and societal perspective of providing reimbursement for psychological treatment for cancer patients with AD.

This study aimed to assess the efficacy and budget impact of the AD‐program among cancer patients with AD. We expected the AD‐program to reduce distress and improve mental adjustment to cancer and health‐related quality of life. The results are relevant to improve psychosocial care including accessibility and reimbursement for cancer patients with AD.

## 2 Methods

### 2.1 Design

A detailed description of the methods and randomisation process can be found in the protocol paper [21]. The RCT was designed comparing two groups (1:1), one with access to the AD‐program and a waitlist control group, with measures before randomisation (T0), and 3 (T3) and 6 months thereafter (T6). Ethical approval was obtained from the Medical Ethical Committee of the VU University Medical Center separately for the recruitment process (2018.524) and the RCT (2019.002).

### 2.2 Population and Inclusion Procedure

From September 2019 to September 2021, patients were recruited via 16 departments of 7 hospitals and one centre for psychosocial cancer care in the Netherlands. Recruitment was halted between March and September 2020 due to the COVID‐19 pandemic. Inclusion criteria were: diagnosed with cancer (except non‐melanoma skin cancer) before July 2018; finished cancer treatment with curative or palliative intent (except endocrine therapy); and aged ≥ 18 years. Participants who provided informed consent for the first part of the study, were screened for increased risk for AD using the Distress Thermometer (DT), the DT problem list [23], and the Hospital Anxiety and Depression Scale (HADS) [24].

The project group initially defined increased risk as: HADS ≥ 11 OR DT ≥ 4 OR wanting to talk to a psychologist or social worker OR work/school/study, family or social problems, emotional problems, fatigue [21]. In a pilot study, we observed that many patients who scored positive on the last criterion (i.e., ‘work/school/study … fatigue’) were not diagnosed with AD. Therefore, increased risk for AD was redefined without this criterion. When the study was put on hold due to the pandemic, we observed that also a proportion of the patients who did not want to talk to a psychologist or social worker, were eventually not diagnosed with AD and/or were not interested in participating in the RCT. To minimise the burden of diagnostic interviews, from a patient perspective and for project resources, the criteria ((HADS‐total ≥ 11 OR DT ≥ 4) AND wanting to talk to a professional) were used from September 2020 to September 2021.

Patients at increased risk for AD were invited for a diagnostic interview by a healthcare psychologist. These psychologists were trained to follow the Dutch guideline, in which AD in cancer patients is defined based on the DSM‐5 criteria and the interaction among three pillars: stressors (e.g., body changes), resilience (e.g., social support), and symptoms and complaints (e.g., fear) [2]. Patients diagnosed with AD were invited to participate in the RCT, except those already receiving psychological treatment (exclusion criterion). After obtaining written informed consent for the second part of the study, patients completed the baseline questionnaire, and were randomised to either the intervention or control group.

### 2.3 Intervention

The AD‐program was designed based on the Dutch guideline [2] and was delivered by a registered healthcare psychologist specialised in psycho‐oncology and trained in the AD‐program. This program consisted of three modules. First, a psychoeducation module (1–4 sessions), if needed, followed by two additional modules (each 1–6 sessions). These additional modules could include one of the evidence‐based interventions outlined in the guideline, such as cognitive behavioural therapy or mindfulness‐based therapy, offered in any format (e.g., individually, in group, online), tailored to the complaints, needs and illness phase of the patient. After each last session of a module, the psychologist and patient evaluated whether a subsequent module was needed.

Patients randomised to the waitlist control group received the AD‐program after a 6‐month waitlist period. During this period, it was allowed to receive psychological care.

### 2.4 Outcome Measures

The primary outcome measure was psychological distress. Secondary outcomes were mental adjustment to cancer and health‐related quality of life. For the budget impact analyses healthcare utilisation, received informal care, and work productivity losses due to absenteeism and presenteeism were assessed.

Psychological distress was measured using the total score of the 14‐item Hospital Anxiety and Depression Scale (HADS) [24]. The HADS has two 7‐item subscales: anxiety and depression. All items refer to the last week. A total score is obtained by summing up the item scores. A higher score indicates a higher level of distress, anxiety or depression. The HADS is a valid instrument for use in Dutch cancer patients.

Adjustment to cancer was measured using the Mental Adjustment to Cancer scale (MAC) [25]. The MAC consists of 40 items, divided over five subscales: fighting spirit, helplessness/hopelessness, anxious preoccupation, fatalism and avoidance. All items refer to the current situation. A higher score indicates more fighting spirit, helplessness/hopelessness, anxious preoccupation, fatalism or avoidance. In addition, two summary scores can be calculated: positive and negative adjustment. The MAC was validated in Dutch cancer patients [25].

Health‐related quality of life was assessed using the EORTC QLQ‐C30 [26], which consists of a global health‐related quality of life scale, five functional scales (physical, role, emotional, cognitive and social functioning), three symptom scales (nausea and vomiting, fatigue and pain) and six single items relating to dyspnoea, insomnia, loss of appetite, constipation, diarrhoea and financial difficulties. All scales and single items are converted to a 0–100 score. A higher score on the global or functioning scales represents a better quality of life, whereas a higher score on the symptom scales or single items indicates a higher level of symptoms. The EORTC QLQ‐C30 is extensively validated in cancer patients.

For the budget impact analyses, healthcare utilisation and received informal care were assessed via the Medical Consumption Questionnaire (iMCQ) [27]. Losses due to absenteeism and presenteeism from work were assessed with the productivity cost questionnaire (iPCQ) [27]. An adapted version of both questionnaires was used with a recall period of 3 months.

Sociodemographic and clinical characteristics were collected via respectively self‐report and the Netherlands Cancer Registry [28]. Usual psychological care received during the study in the control group was assessed using the iMCQ at T3 and T6.

### 2.5 Sample Size

To demonstrate an effect size of 5 points on the HADS as statistically significant, anticipating a standard deviation of 11, 77 participants in each condition were required at follow‐up (power 80%, two‐sided significance level 5%). Anticipating a dropout rate of 25% between baseline and T6, 103 participants per condition, thus 206 in total, needed to be included at baseline. Estimating the willingness to participate in this RCT at 60%, 343 patients diagnosed with AD needed to be approached.

### 2.6 Statistical Analyses Efficacy

Analyses were performed using the IBM Statistical package for the Social Science (SPSS) version 28 (IBM Corp., Armonk, NY USA). Descriptive statistics were generated for all socio‐demographic and clinical characteristics and outcome measures. Chi‐square tests and independent samples *t*‐tests were used to analyse whether randomisation resulted in comparable patient groups.

Analyses were performed according to the intention‐to‐treat principle. A two‐tailed *p*‐value < 0.05 was considered statistically significant. Linear mixed model analyses (LMM) were used to assess the effectiveness of the AD‐program on the course of the primary and secondary outcome measures. LMM contained a fixed effect for group, time and their two‐way interaction, and a random effect for subject. A significant two‐way interaction indicated a difference in effectiveness between the intervention and the control group over time. Furthermore, the reliable change index (RCI) was calculated as the change between the individual's pre (T0) and post (T3 or T6) score divided by the standard error of the difference between the scores ($\sqrt{\left(2{\text{se}}^{2}\right)}$). There is a reliable improvement or deterioration if RCI ≥ 1.96 or RCI ≤ −1.96.

### 2.7 Budget Impact Analyses

To investigate the budget impact, data from this RCT was combined with data from the pre‐post study on the AD‐program [22]. Both studies had the same inclusion and exclusion criteria. The recruitment processes were different. In the pre‐post study, patients were referred to a psychologist by a health care professional in routine care, while in the RCT patients were actively screened in participating hospitals.

The budget impact analyses were conducted in accordance with the principles of good clinical practice of the Professional Society for Health Economics and Outcomes Research (ISPOR) [27] and the Netherlands Organization for Health Research and Development directory [29]. To investigate the budget impact of an intervention, three factors are of importance, namely: (1) the actual size of the population, which in the current study depended on the estimated number of cancer patients diagnosed with AD annually, the percentage of patients who accept referral to the AD‐program, and the available capacity to treat these patients; (2) the costs of the AD‐program compared to current care; and (3) other costs not directly linked to the AD‐program, which may be influenced by offering the AD‐program. Table 1 provides insight into the input parameters for these calculations.

**TABLE 1:** Input parameters of the budget impact analyses.

Parameter	Value	Reference
Size		
Cancer population in the Netherlands		
Number of patients newly diagnosed	119,902	Data from Netherlands Cancer Registry accessed via Kanker.nl: incidence in 2019^a^
Estimated increase per year	2.44%	Data from Netherlands Cancer Registry accessed via Kanker.nl: mean increase in incidence per year in the period 2014–2019^a^
Number of patients living with cancer—5 years prevalence	370,958	Data from Netherlands Cancer Registry accessed via Kanker.nl: 5‐year prevalence (2019)
Eligible patients		
Estimated prevalence rate of AD	15%	van Beek et al. [4]
Estimated acceptance rate	65%	van Beek et al. [4]
Estimated percentage eligible for the intervention	15% * 65% = 9.8%	Calculation based on above presented estimates
Capacity in current clinical practice		
Yearly capacity 2018–2021	123 * 4 quarters = 492	Pre‐post study: Number of new patients treated in the first quarter of 2020 times 4 (i.e., quarter with the highest enrolment)
Capacity year 1	1000	Assumption based on enrolment pre‐post study
Capacity year 2	2000	Assumption * 2 (capacity changes due to more available psychologists)
Capacity year 3	4000	Assumption * 4 (capacity changes due to more available psychologists)
Capacity year 4	8000	Assumption * 8 (capacity changes due to more available psychologists)
Capacity year 5	16,000	Maximum capacity (capacity changes due to more available psychologists)
Costs		
Number of sessions—Estimate 1	10 sessions	Pre‐post study
Number of sessions—Estimate 2	7 sessions	Current RCT
Number of sessions—Estimate 3	16 sessions	Maximum number of sessions following the guideline on adjustment disorder in cancer
Psychological diagnostic interview (2022)	€228,60	Dutch Healthcare Authority tariff 2022
Psychological treatment session (2022)	€192,60	Dutch Healthcare Authority tariff 2022 (60 min)
Other costs		
Healthcare utilisation (iMCQ)		Current RCT, supplement A
Outpatient session	€98	Dutch National Health Care Institute general hospital outpatient clinic indexed to 2022
Hospitalisation day	€541	Dutch National Health Care Institute indexed to 2022
General practitioner visit	€40	Dutch National Health Care Institute indexed to 2022
Absence from work per hour	€41	Dutch National Health Care Institute indexed to 2022
Employment rate	36%	Current RCT

^a^ The incidence as measured in 2019 was used, as the incidence of cancer in 2020 showed a sharp decline (probably due to COVID‐19). The incidence of 2021 and 2022 is not yet completely known and might be an overestimation due to missed cases in the COVID‐19 period.

Size of the study population was based on the prevalence of AD in cancer patients in the Netherlands (15%) and the percentage of cancer patients with AD that wish psychological treatment (65%) [4]. The total potentially eligible population was based on the total number of cancer patients diagnosed with cancer per year (incidence 2019) and the total population living with cancer in the Netherlands in 2019 (5‐year prevalence) [28]. We assumed that after 4 years, the number of cancer patients eligible to participate in the AD‐program will be limited to only a percentage of newly diagnosed patients. Regarding the capacity (i.e., maximum number of patients that can be treated given the total number of healthcare psychologists trained in psycho‐oncology) several scenarios were investigated. The minimum capacity was set at 1000 (i.e., about twice the number of cancer patients diagnosed with AD, treated in the quartile in which the largest number of patients were treated in the pre‐post study, times four). The maximum capacity was set at 16,000 new patients (i.e., 194 psychologists who work on average 30 h per week (the average number of hours employed people work in the Netherlands)).

Costs of the AD‐program were based on three scenario's: (1) the mean number of sessions per patient in the pre‐post study (i.e., 10 sessions); (2) the mean number of sessions in the RCT (i.e., 7 sessions); and (3) the maximum number of 16 sessions. Total costs per session was based on the tariff of the Dutch Healthcare Authority in 2022. To take into account a possible influence on other costs we estimated that: (1) the AD‐program does not have an impact on other costs; (2) that the difference in costs is comparable to the difference in healthcare use (yes/no) (costs were only taken into account in case they significantly differed); (3) that there is an effect, based on previous studies [30, 31] and expert opinion, on respectively outpatient care (assumption: reduction of 2 visits) and hospitalisation (assumption: reduction of 1 hospital day), general practitioner visits (assumption: reduction of 4 visits) and work productivity losses (assumption: reduction in absence from work of 40 h among employed patients).

The budget impact analyses were performed using the budget impact calculation tool of the Netherlands Organization for Health Research and Development [29]. All analyses were performed for a time period of 5 years [27]. In the base case scenario it was estimated that the number of cancer patients treated for AD per year will increase, as the capacity and the referral to psychological treatment is expected to increase over time. The number of sessions was estimated at 10. No effect on other costs were estimated.

Several sensitivity analyses were explored (only changes in parameters in comparison to the base case analyses are discussed), namely (1) a scenario in which the number of sessions is estimated at 7, which also results in a higher treatment capacity; (2) scenarios in which an effect on respectively outpatient care and hospitalisation, (3) visits to the general practitioner, (4) absence from work and (5) a combination of these four categories were expected; (6) a scenario in which there are no capacity problems and all patients can thus be treated; (7) a scenario in which all patients need the maximum of 16 sessions, which also results in a lower treatment capacity; (8) scenarios in which respectively 100%, (9) 80% and (10) 60% of all patients treated for AD were previously also treated, however, under a different diagnosis code (e.g., depression or anxiety disorder).

## 3 Results

### 3.1 Study Population

**FIGURE 1:**
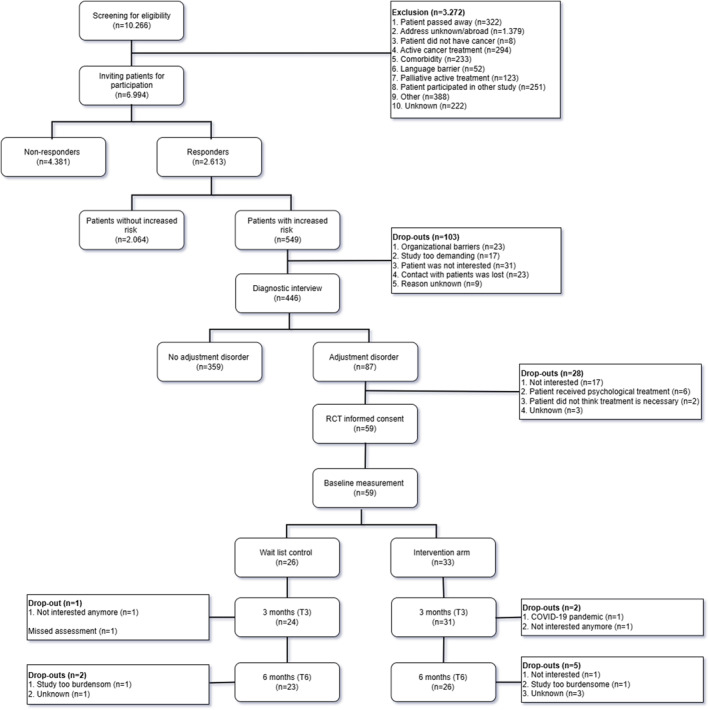
Flow diagram of recruitment process and trial.

The study flow is shown in Figure 1. In total, 10,266 cancer patients were screened for eligibility between September 2019 and September 2021.

In July 2021 we evaluated the inclusion process and observed that 2253 eligible and responding patients were needed to diagnose 58 patients with AD. It was estimated that another 10,000 cancer patients should be screened for their eligibility to include the planned 206 patients in the RCT. This was much higher than the anticipated number of 6238 [21] patients and deemed not to be feasible within an acceptable time frame and reasonable costs. Also, the course of the COVID‐19 pandemic beyond 2021 remained uncertain. Therefore, inclusion of patients ended per September 2021.

In total, 59 out of 93 patients diagnosed with AD participated in the RCT (participation rate 63%), resulting in a sample with insufficient power to detect the anticipated effect size of 5 points on the HADS.

There were no significant differences between patients randomised to the intervention group (*n* = 33) versus the control group (*n* = 26) regarding sociodemographic and clinical characteristics (Table 2) and psychological distress, mental adjustment to cancer and health‐related quality of life as measured at baseline (Table S1). Participants were on average 62 years old, the majority was female, married, diagnosed with stage I or II breast cancer and were diagnosed with cancer longer than 5 years ago.

**TABLE 2:** Overview of the study population in the RCT.

	Total group (*n* = 59)	Intervention (*n* = 33)	Control (*n* = 26)	*p*‐value
	*N* (%)	*N* (%)	*N* (%)	
Age (*M*, SD)	62 (11)	62 (11)	62 (11)	0.79
Gender (female)	38 (64%)	24 (73%)	14 (54%)	0.13
Married (yes)	44 (75%)	25 (76%)	19 (73%)	0.81
Education (high)	20 (34%)	11 (33%)	9 (35%)	0.92
Paid work (yes)	21 (36%)	12 (36%)	9 (35%)	0.89
Tumour site				0.45
Breast	29 (49%)	20 (61%)	9 (35%)	
Prostate	9 (15%)	3 (9%)	6 (23%)	
Head and neck	6 (10%)	3 (9%)	3 (11%)	
Colorectal	4 (7%)	2 (6%)	2 (8%)	
Melanoma	7 (12%)	3 (9%)	4 (15%)	
Lymphoma	2 (3%)	1 (3%)	1 (4%)	
Gynaecological	1 (2%)	0 (0%)	1 (4%)	
Tumour stage				0.16
I and II	43 (73%)	26 (78%)	17 (65%)	
III and IV	13 (22%)	5 (15%)	8 (31%)	
Treatment				0.17
Surgery	19 (32%)	6 (18%)	13 (50%)	
Radiotherapy	4 (7%)	3 (9%)	1 (4%)	
Chemotherapy	1 (2%)	0 (0%)	1 (4%)	
Surgery + radiotherapy	4 (7%)	3 (9%)	1 (4%)	
Surgery + chemotherapy	7 (12%)	6 (18%)	1 (4%)	
Radiotherapy + chemotherapy	0 (0%)	0 (0%)	0 (0%)	
Surgery + radiotherapy + chemotherapy	20 (34%)	12 (36%)	8 (31%)	
Hormone therapy	2 (3%)	1 (3%)	1 (4%)	
No active treatment	1 (2%)	1 (3%)	0 (0%)	
Time since diagnosis (years)				0.83
0–5	17 (29%)	9 (27%)	8 (31%)	
> 5	41 (70%)	23 (70%)	18 (69%)	

*Note:* Missing values: tumour site 1; tumour stage 3; treatment 1; time since diagnosis 1.

### 3.2 Efficacy

Results of the LMM and RCI analyses are provided in Table 3. In the intervention group (*n* = 33) the mean distress score decreased over time (*M* = 19.2, SD = 6.6 at T0, *M* = 16.9, SD = 7.3 at T3 and *M* = 15.6, SD = 7.4 at T6). This decrease was not significantly different from decrease over time in the control group (*n* = 26, *M* = 17.5, SD = 7.2 at T0, *M* = 17.4, SD = 7.7 at T3 and *M* = 15.9, SD = 7.8 at T6, *p* > 0.05). Also, no statistically significant differences between the intervention and control group were found on the course over time of mental adjustment to cancer and health‐related quality of life. In addition, there was no reliable change from baseline to T3 and from baseline to T6 in both conditions (Table S2).

**TABLE 3:** Results of linear mixed model analyses and reliable change index of the course of the primary outcome measure (intention to treat).

	Baseline (T0)		3 months follow‐up (T3)			6 months follow‐up (T6)			*p*‐value
	*N*	Mean (SD)	*N*	Mean (SD)	RCI	*N*	Mean (SD)	RCI	
Distress (HADS‐T)									0.22
Intervention	33	19.2 (6.6)	31	16.9 (7.3)	−0.58	26	15.6 (7.4)	−0.90	
Control	26	17.5 (7.2)	24	17.4 (7.7)	−0.03	23	15.9 (7.8)	−0.49	
Anxiety (HADS‐A)									0.36
Intervention	32	10.4 (3.8)	31	9.3 (4.0)	−0.49	26	8.6 (4.1)	−0.83	
Control	26	9.7 (3.8)	24	9.7 (3.9)	−0.02	23	8.6 (4.2)	−0.61	
Depression (HADS‐D)									0.26
Intervention	32	8.7 (4.4)	31	7.6 (4.3)	−0.46	26	7.0 (4.8)	−0.63	
Control	26	7.8 (4.2)	24	7.7 (5.1)	−0.02	23	7.4 (4.8)	−0.19	

*Note:* RCI: Reliable Change Index, compared to baseline scores. *p*‐value of the interaction between group and time in the LMM analyses.

Of the 33 patients in the intervention group, 29 started the AD‐program (88%), at T3 22 patients (67%) and at T6 12 patients (36%) had not finished the AD‐program yet (Figure S1). Among the patients that started with the AD‐program, the mean number of sessions was 7.3, with a median of 8, and a range of 1–16. In the control group, 3 out of the 26 patients (12%) used psychological care during the study (Table S3). Unintended harms or effects of the AD‐program were not reported.

### 3.3 Budget Impact Analysis

An overview of the budget impact parameters and the base case scenario are presented in Tables 1 and 4. In the base case scenario it was estimated that the number of patients treated for AD per year will increase from 1000 to 14,430 per year, as the capacity for treatment and the referral to the AD‐program will increase over these 5 years. The number of sessions was estimated at 10 [22]. No effect on other costs was estimated, because no differences were found on health care utilisation and absence from work in the RCT.

**TABLE 4:** Calculation of the base case budget impact analysis.

Parameter	2023	2024	2025	2026	2027
Size					
Incidence					
Number of new patients	17,985	18,424	18,873	19,334	19,806
% that wish psychological treatment	65%	65%	65%	65%	65%
Number of potential patients	11,690	11,976	12,267	12,567	12,874
Potential number of patients on the waitlist^a^					
Number of patients	13,911	13,911	13,911	13,911	1
% that wish psychological treatment	65%	65%	65%	65%	65%
Number of potential patients	9042	9042	9042	9042	1
Total size					
Total number of patients	20,732	22,991	23,409	23,550	14,430
Treatment capacity	1000	2000	4000	8000	16,000
Number of patients not treated	19,732	20,991	19,409	15,550	0
% that goes to the waitlist	10%	10%	10%	10%	10%
Number of patients on the waitlist	1973	2099	1941	1555	0
Number of patients treated	1000	2000	4000	8000	14,430
Costs					
Costs AD‐program	1,962,000	3,924,000	7,848,000	15,696,000	28,311,660
Costs psychological treatment control group	0	0	0	0	0
Other costs					
Effect on other costs	0	0	0	0	0
Budget impact (base case)	1,962,000	3,924,000	7,848,000	15,696,000	28,311,660

^a^ Waitlist of patients due to the fact that psychological treatment for adjustment disorder of cancer patients was previously not reimbursed.

The sensitivity analyses resulted in a wide range of budget impact estimates over a time period of 5 years, ranging from no additional costs (in case all patients were already treated under a different diagnosis code) to 191 million euro (in case there are no capacity problems and there is a large waitlist of patients with AD) (Table S4). Likely, however, the budget impact over a time period of 5 years lies between 14 million (in case of a positive effect on other costs) and 58 million (in case of the absence of such an effect). After reaching a stable capacity to treat patients (i.e., 14,430 patients in year 5), the budget impact potentially lies between 7 and 28 million per year.

## 4 Discussion

This study aimed to provide evidence on the efficacy and budget impact of the AD‐program for cancer patients diagnosed with AD. The difference in decrease over time between the intervention and control group was not statistically significant. Also, no significant differences were found for symptoms of depression and anxiety, mental adjustment to cancer and health‐related quality of life. However, these findings should be interpreted with caution, because the RCT was underpowered due to recruitment problems during the COVID‐19 pandemic.

The non‐significant findings in the current RCT contrast with the significant beneficial effects of the AD‐program found in the observational real‐world pre‐post study [22]. In addition to insufficient power in the RCT, there are several other differences, regarding the recruitment procedure and population that may explain these different outcomes. In the RCT, all participants were invited to participate in a scientific study, screened and diagnosed with AD in a standardised diagnostic interview. In the pre‐post study, participants followed the usual route to mental health care. They generally were referred by another health care provider or sought help on their own. This resulted in a population in the RCT that was on average older, longer after diagnosis, and more distressed. Previous research has shown that psychological interventions are generally more effective among patients with more distress [32]. However, participating healthcare psychologists tentatively suggested that patients in the RCT also appeared to be more passive, without a very clearly articulated need for help, while patients in the real‐world pre‐post study seemed to be less distressed, more active and with a more clearly expressed need for help. These differences in recruited patient populations may partially explain these contrasting findings.

The difference between the two studies may also have to do with the different methods used. The pre‐post study had sufficient power, an assessment at baseline and after the intervention, only analysed complete cases, and had no control condition [22]. The RCT was underpowered, had follow‐up assessments while the AD‐program was still ongoing in 36% of the patients, followed the intention‐to‐treat principle, and a control condition in which 12% of the patients received psychological care and improvement also occurred. In the intervention condition of the RCT, the decrease in psychological distress from baseline to 6‐months follow‐up was −3.6, and the decrease in the control group was −1.6. The decrease in the pre‐post study was −5.1. Thus, although distress decreased in both studies and there is ample evidence for the effectiveness of psychological interventions for cancer patients [8, 9, 10, 11, 12, 13, 14, 15, 16, 17], this beneficial effect cannot be confirmed for patients diagnosed with AD by the current RCT.

The budget impact of offering the AD‐program in routine care under various scenarios was estimated at 7 to 28 million euros per year. The budget impact plays a major role in decision‐making and is a key determinant in implementation frameworks. The outcome that the budget impact is most likely between 7 and 28 million euros per year only applies to the Dutch health care system. To put this amount into perspective: the total healthcare costs in 2019 in the Netherlands amounted to 96.9 billion and the total healthcare expenditure for cancer was 6.5 billion [33]. This approach serves as an example for researchers in other countries to perform budget impact analyses of offering the AD‐program to cancer patients with AD.

### 4.1 Limitations

A strength of this study is that the efficacy of the AD‐program was investigated in cancer patients with a confirmed diagnosis of AD. Such studies are scarce. Another strength is that the evaluation of the budget impact adds important knowledge to the literature on economic evaluations of psychological interventions for cancer patients. Limitations include that the study was partially conducted during the COVID‐19 pandemic. In this period the prevalence of AD seemed to have dropped, which hampered the inclusion process and resulted in an underpowered RCT. Another limitation is that the heterogeneous nature of the AD‐program precludes saying anything about the efficacy of the individual interventions offered within this program. A third limitation is that 36% of the patients were still receiving psychological treatment when the last assessment was scheduled. Therefore, the results of this study should be generalised with caution. Limitations of the budget impact analysis are that the results are limited to the Dutch health care system, and that a scenario with the cost of active screening for AD was not included. This scenario was not taken into account, because patients in usual care are usually not actively screened for AD.

### 4.2 Implications

In the future, a sufficiently powered RCT is needed to provide evidence for the AD‐program. Furthermore, research is needed to obtain knowledge on the real world adoption and implementation of the AD‐program in psychosocial cancer care. More research is also needed on the contribution of each module of the AD‐program. The cost‐saving potential of this program is related to the design in which patients are first offered a short module and are only offered another module if the previous one is insufficiently effective.

## 5 Conclusion

In this (underpowered) RCT, the decrease of psychological distress in cancer patients diagnosed with AD receiving the AD‐program, was not statistically different from changes in the control condition. The estimated budget impact of the AD‐program is between 7 and 28 million per year. More research into the efficacy and budget impact of the AD‐program is needed, to come to substantiated findings that can serve as a knowledge base for policymakers.

## Author Contributions

F. J., I. M. V., J. B. P.: funding acquisition. F. J., I. M. V., J. B. P., C. L.: conceptualization. K. H., F. E. B., L. M. A. W., A. M. K.: investigation and project administration. S. E. J. E., I. M. O., J. E. M. W., J. A. W., I. L. E. J., I. H. J. T. H.: resources (patient recruitment). E. J. A., S. V., D. T. B., G. W., I. S.: resources (diagnostic interviews and providing AD‐program). K. H., F. J., V. M. H. C., B. I. L.: data curation and formal analysis. K. H., F. J., I. M. V.: writing – original draft. J. A. E. C., N. M. H., A. M. K., C. L., B. H. R., J. B. P.: writing – review and editing. All authors: writing – final review of the manuscript.

## Ethics Statement

The trial was approved by the Medical Ethics Committee of VU University Medical Center on 03‐06‐2019 (Protocol number 2019.002). All participants provided written informed consent to participate in the trial. The trial protocol is published in the Dutch Trial Register (NL7763) and can be accessed at https://www.trialregister.nl/trial/7763.

This study was conducted according to the principles of the Declaration of Helsinki (version, October 2013) and in accordance with the Dutch Medical Research Involving Human Subjects Act (WMO).

## Conflicts of Interest

The authors declare no conflicts of interest.

## Supporting information

Supporting Information S1

## Data Availability

The full trial protocol can be requested from the corresponding author. Full dataset and statistical code will be made available in a repository after publication of all study outcomes in a peer‐reviewed journal.
