# Supplementary material for: Efficacy and Budget Impact of a Tailored Psychological Intervention Program Targeting Cancer Patients With Adjustment Disorder: A Randomised Controlled Trial
Source: Psychooncology. 2025 Mar 15;34(3):e70123. doi: 10.1002/pon.70123 (PMC11910135; doi:10.1002/pon.70123)
Supplement: Supplementary file 1 — Supporting Information S1 [file PON-34-e70123-s001.docx]

**Supporting Information: Supplementary tables and figures**

**Supplementary Table 1**

*Baseline scores on outcome measures*

|  | Total group  (n = 59) | Intervention  (n = 33) | Control  (n = 26) |  |
| --- | --- | --- | --- | --- |
|  | M (SD) | M (SD) | M (SD) | P-value |
| Distress (HADS-T) | 18.4 (6.8) | 19.2 (6.6) | 17.5 (7.2) | .36 |
| Anxiety (HADS-A) | 10.1 (3.8) | 10.4 (3.8) | 9.7 (3.8) | .49 |
| Depression (HADS-D) | 8.3 (4.3) | 8.7 (4.4) | 7.8 (4.2) | .40 |
| Mental adjustment to cancer (MAC) |  |  |  |  |
| Fighting spirit | 44.4 (5.8) | 44.5 (5.7) | 44.2 (6.0) | .86 |
| Helpless/hopeless | 13.3 (3.9) | 13.3 (3.7) | 13.2 (4.3) | .89 |
| Anxious preoccupation | 22.6 (4.0) | 22.5 (4.5) | 22.8 (3.5) | .76 |
| Fatalism | 19.6 (3.6) | 19.9 (3.8) | 19.2 (3.4) | .44 |
| Avoidance | 1.7 (0.8) | 1.7 (0.8) | 1.8 (1.0) | .75 |
| Health-related quality of life (EORTC QLQ-C30) | | |  |  |
| Global quality of life | 60.0 (19.6) | 58.3 (17.7) | 62.2 (21.9) | .46 |
| Physical functioning | 74.8 (20.7) | 70.5 (21.1) | 80.3 (19.2) | .07 |
| Role functioning | 62.1 (31.2) | 60.6 (29.7) | 64.1 (33.6) | .67 |
| Emotional functioning | 54.0 (22.4) | 53.0 (22.2) | 55.1 (23.0) | .72 |
| Cognitive functioning | 57.9 (27.9) | 55.6 (25.2) | 60.9 (31.2) | .47 |
| Social functioning | 67.5 (30.9) | 67.7 (32.8) | 67.3 (28.9) | .96 |
| Fatigue | 49.0 (29.1) | 53.5 (30.2) | 43.2 (27.1) | .18 |
| Nausea/vomiting | 13.3 (24.7) | 15.2 (25.8) | 10.9 (23.5) | .52 |
| Pain | 35.3 (31.3) | 39.4 (31.7) | 30.1 (30.6) | .26 |
| Dyspnoea | 28.2 (30.2) | 32.3 (31.7) | 23.1 (27.9) | .25 |
| Insomnia | 50.3 (32.4) | 52.5 (32.3) | 47.4 (32.9) | .55 |
| Loss of appetite | 19.8 (31.0) | 22.2 (36.0) | 16.7 (23.6) | .50 |
| Constipation | 19.8 (29.8) | 18.2 (29.0) | 21.8 (31.2) | .65 |
| Diarrhoea | 15.3 (28.6) | 18.2 (31.3) | 11.5 (24.8) | .38 |
| Financial problems | 13.6 (27.1) | 17.2 (29.0) | 9.0 (24.1) | .25 |

**Supplementary Table 2**

*Results of linear mixed model analyses and reliable change index of the course of the secondary outcome measures (intention to treat)*

|  |  | Baseline (T0) | | | 3 months follow-up (T3) | | | 6 months follow-up (T6) | | | |  |
| --- | --- | --- | --- | --- | --- | --- | --- | --- | --- | --- | --- | --- |
|  |  | N | Mean (SD) | | N | Mean (SD) | RCI | N | | Mean (SD) | RCI | P-value |
| Mental adjustment to cancer (MAC) | | | | |  | | |  |  | | |  |
| Fighting spirit | | | | |  | | |  |  | | | 0.17 |
|  | Intervention | 33 | 44.5 (5.7) | | 31 | 45.0 (4.2) | 0.06 | 26 | | 45.4 (4.9) | 0.05 |  |
|  | Control | 26 | 44.2 (6.0) | | 24 | 42.9 (5.3) | -0.28 | 23 | | 44.9 (5.0) | 0.08 |  |
| Helpless/hopeless | | | |  |  | | |  |  | | | 0.93 |
|  | Intervention | 33 | 13.3 (3.7) | | 31 | 12.7 (3.4) | -0.32 | 26 | | 12.2 (4.0) | -0.57 |  |
|  | Control | 26 | 13.2 (4.3) | | 24 | 12.6 (3.6) | -0.21 | 23 | | 12.3 (3.6) | -0.54 |  |
| Anxious preoccupation | | | | |  | | |  |  | | | 0.74 |
|  | Intervention | 33 | 22.5 (4.5) | | 29 | 21.4 (3.7) | -0.29 | 26 | | 21.4 (3.8) | -0.22 |  |
|  | Control | 26 | 22.8 (5.3) | | 24 | 22.2 (3.2) | -0.18 | 23 | | 22.1 (2.8) | -0.34 |  |
| Fatalism | | | |  |  | | |  |  | | | 0.59 |
|  | Intervention | 33 | 19.9 (3.8) | | 31 | 18.5 (2.9) | -0.39 | 26 | | 18.9 (3.4) | -0.33 |  |
|  | Control | 26 | 19.2 (3.4) | | 23 | 18.4 (3.9) | -0.14 | 21 | | 18.5 (2.4) | -0.29 |  |
| Avoidance | | | |  |  | | |  |  | | | 0.55 |
|  | Intervention | 33 | 1.7 (0.8) | | 31 | 1.4 (0.6) | n/a | 26 | | 1.5 (0.6) | n/a |  |
|  | Control | 26 | 1.8 (1.0) | | 24 | 1.7 (0.9) | n/a | 23 | | 1.7 (0.8) | n/a |  |
| Health-related quality of life (EORTC-QLQ C-30) | | | | | | | |  |  | | |  |
| Global quality of life | | | | |  | | |  |  | | | 0.59 |
|  | Intervention | 33 | 58.3 (17.7) | | 31 | 59.1 (16.1) | 0.40 | 25 | | 64.2 (19.7) | 0.49 |  |
|  | Control | 26 | 62.2 (21.9) | | 24 | 63.3 (23.1) | 0.03 | 23 | | 64.5 (17.7) | 0.15 |  |
| Physical functioning | | | |  |  | | |  |  | | | 0.42 |
|  | Intervention | 33 | 70.5 (21.1) | | 31 | 75.9 (19.1) | 0.36 | 25 | | 72.9 (19.1) | 0.19 |  |
|  | Control | 26 | 80.3 (19.2) | | 24 | 82.6 (20.4) | 0.09 | 23 | | 79.3 (21.3) | 0.02 |  |
| Role functioning | | | |  |  | | |  |  | | | 0.52 |
|  | Intervention | 33 | 60.6 (29.7) | | 31 | 62.4 (29.6) | 0.09 | 25 | | 64.2 (27.2) | 0.21 |  |
|  | Control | 26 | 64.1 (33.6) | | 24 | 71.4 (26.3) | 0.36 | 23 | | 64.8 (30.7) | 0.03 |  |
| Emotional functioning | | | | |  | | |  |  | | | 0.84 |
|  | Intervention | 33 | 53.0 (22.2) | | 31 | 58.9 (23.8) | 0.43 | 25 | | 59.8 (27.7) | 0.57 |  |
|  | Control | 26 | 55.1 (23.0) | | 24 | 62.6 (24.1) | 0.53 | 23 | | 64.7 (20.9) | 0.78 |  |
| Cognitive functioning | | | |  |  | | |  |  | | | 0.41 |
|  | Intervention | 33 | 55.6 (25.2) | | 31 | 62.1 (29.5) | 0.33 | 25 | | 57.5 (29.4) | -0.08 |  |
|  | Control | 26 | 60.9 (31.2) | | 24 | 69.2 (27.8) | 0.28 | 23 | | 70.4 (27.2) | 0.40 |  |
| Social functioning | | | |  |  | | |  |  | | | 0.28 |
|  | Intervention | 33 | 67.7 (32.8) | | 31 | 69.4 (30.0) | 0.14 | 25 | | 69.2 (27.9) | 0.19 |  |
|  | Control | 26 | 67.3 (28.9) | | 24 | 78.3 (26.9) | 0.54 | 23 | | 67.7 (30.6) | -0.04 |  |
| Fatigue | | | | |  | | |  |  | | | 0.41 |
|  | Intervention | 33 | 53.5 (30.2) | | 31 | 50.7 (26.9) | -0.18 | 25 | | 45.6 (28.8) | -0.59 |  |
|  | Control | 26 | 43.2 (27.1) | | 24 | 38.9 (27.2) | -0.18 | 23 | | 41.1 (27.7) | -0.16 |  |
| Nausea/vomiting | | | |  |  | | |  |  | | | 0.76 |
|  | Intervention | 33 | 15.2 (25.8) | | 31 | 8.6 (17.1) | -0.33 | 25 | | 10.8 (24.0) | -0.25 |  |
|  | Control | 26 | 10.9 (23.5) | | 24 | 7.8 (17.7) | -0.16 | 23 | | 6.4 (17.3) | -0.22 |  |
| Pain | | | |  |  | | |  |  | | | 0.88 |
|  | Intervention | 33 | 39.4 (31.7) | | 31 | 37.3 (33.4) | -.03 | 25 | | 31.3 (28.0) | -0.24 |  |
|  | Control | 26 | 30.1 (30.6) | | 24 | 31.5 (34.2) | 0.13 | 23 | | 22.0 (30.9) | -0.36 |  |
| Dyspnoea | | | | |  | | |  |  | | | 0.27 |
|  | Intervention | 33 | 32.3 (31.7) | | 31 | 23.7 (32.4) | n/a | 25 | | 26.1 (33.3) | n/a |  |
|  | Control | 26 | 23.1 (27.9) | | 24 | 23.6 (31.5) | n/a | 23 | | 17.8 (28.0) | n/a |  |
| Insomnia | | | |  |  | | |  |  | | | 0.61 |
|  | Intervention | 33 | 52.5 (32.3) | | 31 | 49.4 (32.1) | n/a | 25 | | 43.8 (34.0) | n/a |  |
|  | Control | 26 | 47.4 (32.9) | | 24 | 49.5 (31.1) | n/a | 23 | | 47.4 (34.6) | n/a |  |
| Loss of appetite | | | | |  | | |  |  | | | 0.96 |
|  | Intervention | 33 | 22.2 (36.0) | | 31 | 25.0 (31.9) | n/a | 25 | | 21.3 (33.4) | n/a |  |
|  | Control | 26 | 16.7 (23.6) | | 24 | 17.2 (24.0) | n/a | 23 | | 14.6 (26.3) | n/a |  |
| Constipation | | | |  |  | | |  |  | | | 0.36 |
|  | Intervention | 33 | 18.2 (29.0) | | 31 | 18.6 (28.3) | n/a | 25 | | 17.1 (33.5) | n/a |  |
|  | Control | 26 | 21.8 (31.19) | | 24 | 14.1 (31.1) | n/a | 23 | | 12.6 (28.1) | n/a |  |
| Diarrhoea | | | |  |  | | |  |  | | | 0.25 |
|  | Intervention | 33 | 18.2 (31.3) | | 31 | 13.7 (22.2) | n/a | 25 | | 21.7 (28.9) | n/a |  |
|  | Control | 26 | 11.5 (24.8) | | 24 | 14.1 (25.9) | n/a | 23 | | 14.3 (26.3) | n/a |  |
| Financial problems | | | | |  | | |  |  | | | 0.99 |
|  | Intervention | 33 | 17.2 (29.0) | | 31 | 14.0 (24.0) | n/a | 25 | | 14.4 (21.5) | n/a |  |
|  | Control | 26 | 8.9 (24.1) | | 24 | 6.5 (17.0) | n/a | 23 | | 6.6 (17.3) | n/a |  |

*Note*. RCI: Reliable Change Index, compared to baseline scores. P-value of the interaction between group and time in the LMM analyses. n/a not applicable (single-item subscales).

**Supplementary Figure 1**

*Schematic overview of therapy sessions per patient in the intervention group in relation to the outcome assessment times at baseline and 3 and 6 months follow-up*


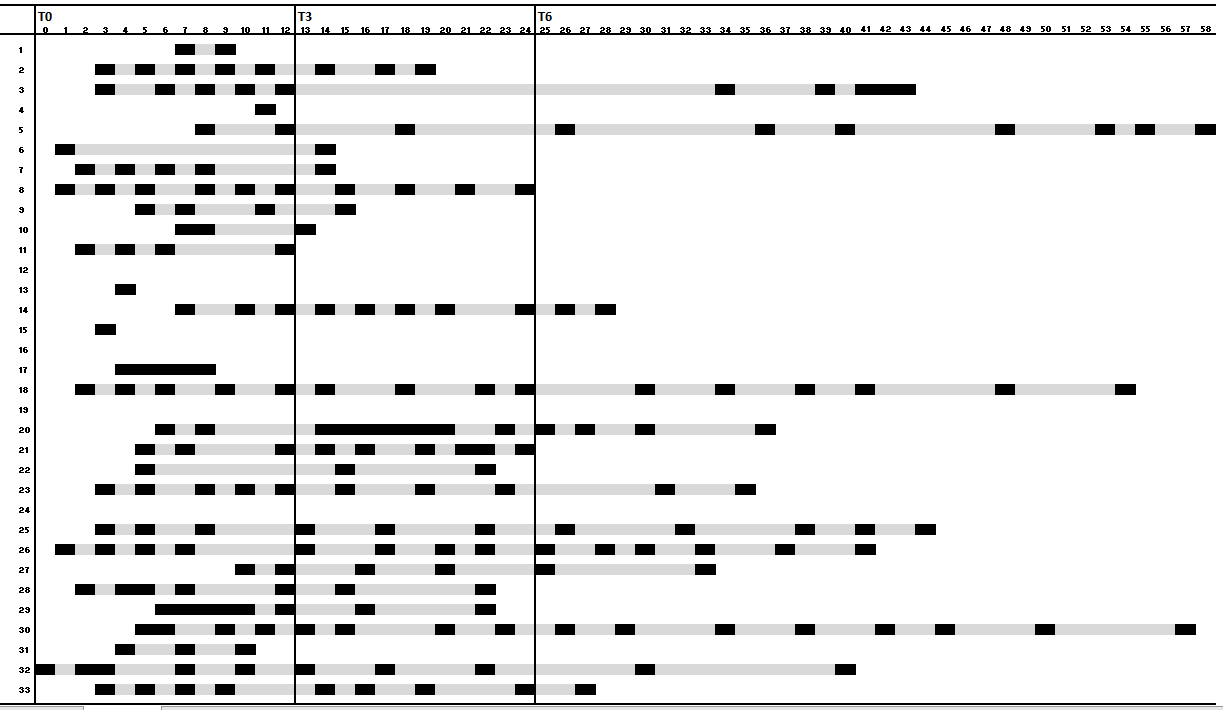


*Note*. Black: week with treatment session. Grey: week between treatment sessions.

**Supplementary Table 3**

*Healthcare utilization and absence from work in the intervention and control group of the RCT*

|  | Baseline (N=59) | | | 3 months follow-up (N=55) | | | 6 months follow-up (N=48) | | |
| --- | --- | --- | --- | --- | --- | --- | --- | --- | --- |
|  | Control  N (%) | Intervention  N (%) | p-value^1^ | Control  N (%) | Intervention  N (%) | p-value^†^ | Control  N (%) | Intervention  N (%) | p-value^†^ |
| GP (telephone) | 10 (38%) | 17 (52%) | 0.32 | 7 (29%) | 14 (45%) | 0.23 | 12 (52%) | 12 (48%) | 0.77 |
| GP (home or practice visit) | 16 (62%) | 23 (70%) | 0.51 | 11 (46%) | 16 (52%) | 0.67 | 13 (57%) | 15 (60%) | 0.81 |
| Social worker | 0 (0%) | 1 (3%) | 1.00 | 2 (8%) | 0 (0%) | 0.19 | 0 (0%) | 1 (4%) | 1.00 |
| Physiotherapist | 8 (31%) | 17 (52%) | 0.11 | 7 (29%) | 14 (45%) | 0.23 | 8 (35%) | 14 (56%) | 0.14 |
| Dietitian | 2 (8%) | 1 (3%) | 0.58 | 3 (13%) | 3 (10%) | 1.00 | 2 (9%) | 1 (4%) | 0.60 |
| Psychologist | n/a^2^ | n/a^‡^ |  | 2 (8%) | n/a |  | 2 (9%) | n/a |  |
| Medical specialist | 17 (65%) | 17 (52%) | 0.28 | 14 (58%) | 12 (39%) | 0.15 | 12 (52%) | 13 (54%) | 0.89 |
| Alternative medicine | 2 (8%) | 6 (18%) | 0.24 | 4 (17%) | 4 (13%) | 1.00 | 3 (43%) | 4 (57%) | 0.63 |
| Hospitalization | 1 (4%) | 0 (0%) | 0.44 | 1 (4%) | 1 (3%) | 1.00 | 2 (9%) | 1 (4%) | 0.60 |
| Care at home (nursing, home care) | 2 (8%) | 1 (3%) | 0.58 | 1 (4%) | 2 (7%) | 1.00 | 1 (4%) | 1 (4%) | 1.00 |
| Informal care | 4 (16%) | 12 (36%) | 0.09 | 5 (22%) | 9 (30%) | 0.50 | 8 (35%) | 11 (44%) | 0.57 |
| Absence from work^§^ | 1 (11%) | 2 (20%) | 1.00 | 2 (29%) | 3 (33%) | 1.00 | 1 (14%) | 0 (0%) | 0.47 |

*Note*. ^†^Chi square or Fisher’s exact test. ^‡^All patients had a diagnostic interview before start of the randomized controlled trial. ^§^Among those who work (i.e. 9 patients in the control group and 10 patients in the intervention group at baseline).

**Supplementary Table 4**

*Overview of the various budget impact scenarios*

| **Scenarios** | **2023** | | | **2024** | | | **2025** | | | **2026** | | | **2027** | | | **Total** | |
| --- | --- | --- | --- | --- | --- | --- | --- | --- | --- | --- | --- | --- | --- | --- | --- | --- | --- |
|  | Patients treated (N) | Budget impact  x €1,000 | Patients treated (N) | | Budget impact  x €1,000 | Patients treated (N) | | Budget impact  x €1,000 | Patients treated (N) | | Budget impact  x €1,000 | Patients treated (N) | | Budget impact  x €1,000 | Patients treated (N) | | Budget impact  x €1,000 |
| Base case | 1,000 | 1,962 | 2,000 | | 3,924 | 4,000 | | 7,848 | 8,000 | | 15,696 | 14,430 | | 28,312 | 29,430 | | 57,742 |
| Scenario with on average 7 sessions | 1,429 | 1,979 | 2,857 | | 3,957 | 5,714 | | 7,914 | 11,429 | | 15,829 | 14,069 | | 19,485 | 35,498 | | 49,164 |
| Scenario with effect on other costs: outpatient, hospitalization | 1,000 | 1,225 | 2,000 | | 2,450 | 4,000 | | 4,900 | 8,000 | | 9,800 | 14,430 | | 17,677 | 29,430 | | 36,051 |
| Scenario with effect on other costs: GP visits | 1,000 | 1,802 | 2,000 | | 3,604 | 4,000 | | 7,208 | 8,000 | | 14,416 | 14,430 | | 26,003 | 29,430 | | 53,033 |
| Scenario with effect on other costs: work | 1,000 | 1,372 | 2,000 | | 2,744 | 4,000 | | 5,488 | 8,000 | | 10,976 | 14,430 | | 19,798 | 29,430 | | 40,378 |
| Scenario with effect on other costs: all of the above | 1,000 | 475 | 2,000 | | 950 | 4,000 | | 1,900 | 8,000 | | 3,800 | 14,430 | | 6,854 | 29,430 | | 13,979 |
| Scenario where all patients are treated | 20,732 | 40,676 | 21,018 | | 41,237 | 21,310 | | 41,810 | 21,609 | | 42,397 | 12,875 | | 25,261 | 97,544 | | 191,381 |
| Scenario with the maximum of 16 sessions | 625 | 1,949 | 1,250 | | 3,898 | 2,500 | | 7,795 | 5,000 | | 15,590 | 10,000 | | 31,180 | 19,375 | | 60,411 |
| Scenario in which all patients  previously received treatment | 1,000 | 0 | 2,000 | | 0 | 4,000 | | 0 | 8,000 | | 0 | 14,430 | | 0 | 29,430 | | 0 |
| Scenario in which 80% previously received treatment | 1,000 | 393 | 2,000 | | 786 | 4,000 | | 1,572 | 8,000 | | 3,144 | 14,430 | | 5,670 | 29,430 | | 11,566 |
| Scenario in which 60% previously received treatment | 1,000 | 785 | 2,000 | | 1,570 | 4,000 | | 3,140 | 8,000 | | 6,280 | 14,430 | | 11,328 | 29,430 | | 23,102 |
